# An Adsorptive and Antioxidant Vaginal Gel Clears High-Risk HPV- and p16/Ki-67-Associated Abnormal Cytological Cervical Findings: A *post-hoc* Subgroup Analysis of a Prospective Randomized Controlled Trial on CIN2 and p16 Positive CIN1

**DOI:** 10.3389/fmed.2021.645559

**Published:** 2021-05-25

**Authors:** Attila Louis Major, Ales Skřivánek, Etienne Marc Grandjean, Vladimír Dvořák, Tomáš Malík, Marek Pluta, Ivanna Mayboroda

**Affiliations:** ^1^Femina Gynaecology Centre, Geneva, Switzerland; ^2^Department of Obstetrics and Gynaecology, Cantonal Hospital, University of Fribourg, Fribourg, Switzerland; ^3^G-CENTRUM Olomouc, s.r.o., Olomouc, Czechia; ^4^Phidalsa Pharma Consultants, Geneva, Switzerland; ^5^Camara and Partners Consultancy, Nyon, Switzerland; ^6^Centrum ambulantní gynekologie a primární péče, s.r.o., Brno, Czechia; ^7^Gyneko spol. s.r.o., Vsetin, Czechia; ^8^Department of Obstetrics and Gynaecology, Fakultní nemocnice v Motole (University Hospital Motol) Onkogynekologická a kolposkopická ambulance, Praha, Czechia; ^9^Department of Obstetrics and Gynaecology, University Hospital of Geneva, Geneva, Switzerland

**Keywords:** cervical intraepithelial neoplasia, abnormal cervical smear findings, high risk HPV, p16/Ki-67 biomarker, adsorptive and antioxidative vaginal gel, silicon dioxide, sodium selenite, tumor environment

## Abstract

**Objective:** To analyze the course of p16/Ki-67-positive abnormal cytological cervical findings and high risk (hr)-HPV- and p16/Ki-67-clearances in women treated with a vaginal gel.

**Methods:** 172 women with a histological diagnosis of CIN2 or p16-positive CIN1 lesions were selected based on a positive cytological p16/Ki-67 test. For 3 months, 75 patients in the active arm (AA) daily administered 5 ml of a vaginal gel. Ninety seven patients in the control arm (CA) underwent no treatment (“watchful waiting”). Endpoints were cytological evolution, p16/Ki-67- and hr-HPV-clearances.

**Results:** At 3 months, cytological regression was observed in 76% (57/75) of patients in the AA compared with 25% (24/97) in the CA. Progression occurred in 5% (4/75) of the AA compared with 15% (15/97) of the CA. The p16/Ki-67 status change was statistically significantly (*p* < 0.001) in favor of the AA: 77% (58/75) became negative compared to 21% (20/97) in the CA. hr-HPV prevalence decreased significantly (*p* < 0.001) in the AA from 87 to 44%, while increasing in the CA from 78 to 84%. Cytological regression and p16/Ki-67 changes persisted in the AA at 6 months.

**Conclusions:** The vaginal gel significantly cleared hr-HPV and p16/Ki-67 and was associated with improved cytological findings, thereby potentially offering an effective option against oncogenic risk.

**Clinical Trial Registration:** Identifier: [ISRCTN11009040].

## Introduction

Cervical cancer remains a major cause of oncologic death in women in many developing countries. In contrast, it is now rare in developed countries and mainly observed in postmenopausal women. Regular cervical screening, based on cervical cytology, is still being recommended in all countries. When there is a positive or suspicious finding, cervical biopsy is required under colposcopic control. By selecting lesions early on for oncogenic transformation with a biomarker, such as p16/Ki-67, it is judicious to propose a preventive treatment during the watch and wait period before repeating the cervical smear or colposcopy. Watch and wait without therapeutic intervention is still part of all gynecological guidelines. However, this is unsatisfactory for both patients and gynecologists. This therapy-free interval is associated with considerable anxiety for patients, occasionally producing a high level of psychological stress (1). While increasing the remission rate of the abnormal findings would lower mental stress, there is still no single validated, non-surgical therapeutic approach for mild-to-moderate cervical intraepithelial neoplasia (CIN).

To date attempts to effectively treat human papilloma virus (HPV)-related low-grade lesions with a non-destructive method have failed, due to adverse events (imiquimod, interferon) or unsatisfactory responses [green tea, metronidazole-containing gel, 5-fluorouracil (5-FU) vaginal cream] (2–4). More invasive interventions may be responsible of serious sequelae, such as dyspareunia, cervical stenosis, and premature delivery, which calls for new guidelines proposing more conservative approaches during this time (5, 6). Until HPV vaccines are universally adopted, the watch and wait period offers the opportunity for administering a non-destructive treatment potentially promoting regression of lesions.

The vaginal gel used in this study (SAM vaginal gel) is based on an aqueous hydroxyethyl cellulose matrix containing highly-dispersed silicon dioxide as well as DEFLAMIN®, a combination of sodium selenite and citric acid, a globally patented formula with powerful antioxidative properties (7). Its primary mechanism of action is based on the adsorptive binding properties of homogenously suspended, micronized silicon dioxide particles. Highly dispersed silicon dioxide is a well-established pharmacologically inert, adsorbent agent. Numerous publications confirm the adsorptive binding of proteins, lipids and lipoproteins, viruses and bacteria by silicon dioxide (8, 9).

Oxidative stress induced by infections and inflammation has an important role in DNA damage and cervical tumorigenesis (10). Studies suggest that oxidative stress likely plays a major role in the process of HPV DNA integration, which is an important step for malignant transformation of the cervical epithelium (11, 12). It has been demonstrated that oxidative DNA damage is a multistep process and the level of damage increases from CIN1 to CIN3 compared to normal epithelium (13). Consequently, early treatment with this vaginal gel may prevent cervical lesions to progress to a higher grade.

We have recently performed a randomized, controlled trial with SAM vaginal gel on colposcopical, histological, and cytological examinations, in which one major inclusion criterion was p16 immunohistochemistry, which has been recently recognized as a valuable, high-specificity prognostic factor (14–16). That study demonstrated efficacy and safety of this particular vaginal gel (14). Most patients were also tested for p16/Ki-67 in cervical smears, recognized for greater specificity than both cytology and HPV (14, 17–21). The presented *post-hoc* analysis was limited to those patients (172 of 216 in the parent trial) who had a positive p16/Ki-67 test at study entry. A specific analysis of the cervical smear data is presented. The analysis aims at comparing the various cervical smear screening tools, in particular the p16/Ki-67 test, but also standard cytology (Bethesda) and hr (high risk)-HPV identification. Further, the subanalysis assesses whether the treatment was effective when only cytological findings are used, including p16/Ki-67. It is anticipated to provide insight into the potential and suitability of these variables as endpoints to establish the beneficial effect of topical treatments on the development of precancerous cervical lesions.

## Methods

Two hundred and sixteen patients were block-randomized (108 in each arm) 1:1 to the SAM vaginal gel arm or “watch and wait” (control) arm. SAM gel was allocated to patients based on randomization lists. Among 216 patients evaluated in the parent prospective randomized controlled trial with histologically proven CIN2 and p16 positive CIN1 (14), 172 patients with positive cytological p16/Ki-67 were selected retrospectively. The clinical investigation included a 3-month treatment period and 3-months of follow-up. In the active arm, treatment comprised 3 × 28-day intravaginal self-administration of SAM gel containing 10.0 mg of highly dispersed silicon dioxide, 24.8 mg of citric acid, and 0.25 mg of selenium per administration (5 ml). The vaginal gel was administered daily, deeply inside the vagina using a single-use applicator. The investigational device was provided by the sponsor DEFLAMED International s.r.o., Prague, Czech Republic.

Patients in the control arm were followed-up with the watch and wait strategy, without any treatment, in compliance with current international guidelines.

Selection criteria for the subgroup analysis of the prospective main study were women with a positive cytological p16/Ki-67 test associated with cytological findings, Atypical Squamous Cells of Undetermined Significance (ASC-US), Low-grade Squamous Intraepithelial Lesion (LSIL), Atypical Squamous Cells cannot exclude HSIL (ASC-H) or High-grade Squamous Intraepithelial Lesion (HSIL). Patients were aged 25-60 years; they all had a histological diagnosis of CIN2 or p16-positive CIN1 at baseline, regardless of HPV findings. All subjects provided their written informed consent, were not pregnant and used a suitable method of contraception during treatment if they were of childbearing age.

Exclusion criteria were a history of oncological or immunological disease, chronic viral disease, including hepatitis, immunosuppressive treatment, pregnancy or breastfeeding, known allergy to the gel or its components, a colposcopy finding suspicious of invasive disease, simultaneous participation at another clinical trial; and for CIN2 patients, unsatisfactory colposcopy (i.e., the transformation zone and/or the lesion were not fully visible); and for CIN1 patients, risk discrepancy with a cytological finding (HSIL).

Cytological samples underwent usual screening analysis, were stained according to Papanicolaou and evaluated in accordance with the Bethesda system. The same sample underwent immunocytochemistry with dual biomarker technology CINtec® PLUS Cytology, Roche. Two independent experts in cervical histo- and cytopathology were involved in the classification of the lesions and in the immune-, histo- and cytochemical analyses. They were blinded to treatment arms and sample materials were identified by patient number only.

Cervical smear samples were also collected for hr-HPV status. Cobas® 4800 HPV Test (Roche Diagnostics, GmbH, Mannheim, Germany) was used to identify 14 genotypes of hr-HPV DNA, with separate genotyping of 16 and 18 hr-HPV and others (31, 33, 35, 39, 45, 51, 52, 56, 58, 59, 66, 68).

The endpoint of this subgroup analysis was cytological regression (change to a lower grade of lesion) or remission (complete healing) after 3 months of using SAM vaginal gel or solely watching and waiting. Success was regarded as either cytological regression (an initial ASC-US, LSIL, ASC-H, or HSIL lesion that disappeared or changed to a lower level e.g., LSIL to ASC-US, etc.) or failed progression. This was assessed 3 months after the start of treatment, and after a further 3-month follow-up. The findings were classified by decreasing risk of squamous cell carcinoma, according to Bethesda: HSIL, ASC-H, LSIL, ASC-US. Another major endpoint was cytological progression from low risk (ASC-US, LSIL) to high risk (ASC-H and HSIL). Two secondary endpoints were considered: cytological change in p16/Ki-67 (CINtec® Plus test) after 3 and 6 months; and clearance of hr-HPV strains at 3 months (22).

Statistical analysis was performed using IBM SPSS Statistics for Windows, Version 25.0. Armonk, NY: IBM Corp. Fisher's exact test with two-tailed *p*-value was used to compute the significance of association between treatments and improvement of cytological findings, HPV clearance, and p16/Ki-67 outcome. Microsoft Office Excel was used for descriptive data evaluation.

## Results

**Figure 1:**
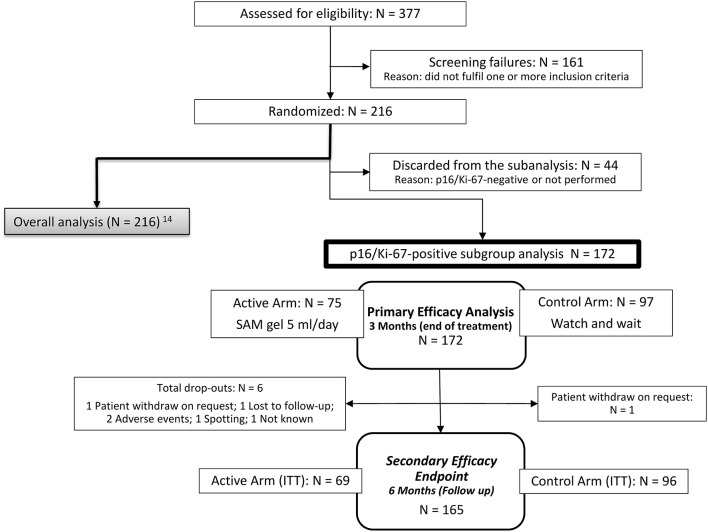
Consort flow diagram showing the participant flow of the 172 patients with p16/Ki-67-positive CIN1 and CIN2. ITT, Intention-to-treat population.

Overall, 172 of 216 patients of the main randomized trial (where 377 patients were screened between May 9, 2017 and July 29, 2018), including all CIN1 and all CIN2, who had a p16/Ki-67-positive test result and were retrospectively selected for the subgroup analysis. Seventy five patients in the active arm and 97 in the control arm fulfilled the selection criteria (Figure 1).

Baseline characteristics are presented in Table 1. Several demographic data differences could be seen between treatment and control arms. Stratification of the patients, according to CIN grade, was not planned in the main trial protocol. For this reason, and because of selection by p16/Ki-67, the distribution of CIN1 and CIN2 patients is different between active and control arms. Biopsies revealed CIN2 to be significantly more frequent in the active arm. The distribution of cytological findings, however, was comparable between both arms (Table 1).

**Table 1:** Baseline characteristics of p16/Ki-67-positive CIN1 and CIN 2 patients (*N* = 172).

**Intention-To-Treat (ITT) population**		**Active arm (*****n*** **=** **75)**		**Control arm (*****n*** **=** **97)**		***P***
Age (years) Mean ± SD		33.32	± 6.86	35.58	± 8.81	0.117^**+**^
Relevant gynecological history^§^		3	4%	17	18%	0.007^**x**^
Smokers		22	29%	28	29%	1.000^x^
HPV vaccination		12	16%	14	14%	0.832^x^
Histology^#^	CIN1	38	51%	86	89%	<0.001^**X**^
	CIN2	37	49%	11	11%	
	Total	75	100%	97	100%	
Cytology	ASC-US	17	23%	20	21%	0.235*
	LSIL	40	53%	57	59%	
	ASC-H	8	11%	15	15%	
	HSIL	10	13%	5	5%	
	Total	75	100%	97	100%	
High-risk HPV^S^	Yes	65	87%	76	78%	0.169^x^
	No	10	13%	21	22%	
	Total	75	100%	97	100%	
CINtec® Plus p16 pos. /Ki-67^SS^	CIN1	38	51%	86	89%	<0.001^**x**^
	CIN2	37	49%	11	11%	
	Total	75	100%	97	100%	
IHC p16^SSS^	CIN1	33/38	87%	44/86	51%	<0.001*
	CIN2	37/37	100%	11/11	100%	
	Total	70/75	93%	48/97	49%	
High-risk HPV	CIN1	31/38	82%	65/86	76%	n.a.
	CIN2	34/37	92%	11/11	100%	
	Total	65/75	87%	76/97	78%	
	Values given as mean ± standard deviation, %. Statistical analyses by ^+^Wilcoxon rank-sum test. ^X^Fisher's two-tailed exact test. *Pearson chi-squared test. ^S^according to HPV Cobas® 4800 test. ^SS^according to CINtec® Plus (p16/Ki-67). ^SSS^according to CINtec® (p16) Histology-Test. IHC p16, ImmunoHistoChemistry p16 (Histology). CINtec® Plus, Immunocytochemistry p16/Ki-67 (Cytology).			^#^CIN1 p16 positive (IHC or CINtec® Plus). n.a. not analyzed. ^§^Relevant gynecological history (conservative surgeries of the uterus and surgeries for adnexal diseases). ASC-US, Atypical Squamous Cells of Undetermined Significance. LSIL, Low-grade Squamous Intraepithelial Lesion. ASC-H, Atypical Squamous Cells cannot exclude HSIL. HSIL, High-grade squamous intraepithelial lesion. Due to rounding, numbers presented may not add up precisely to the totals provided.		

There were significantly more IHC p16 positive patients in the active arm at baseline (Table 1).

After 3 months of treatment, cytological regression and remission were statistically significantly higher (*p* < 0.001) in the active arm (57/75 patients or 76%) than in the control arm (24/97 patients or 25%). Only 4/75 patients (5%) in the active arm progressed to a higher grade of cytological finding, whereas in the control arm 15/97 patients (15%) progressed to a higher grade of cytological finding (Table 2).

**Table 2:** Cytological findings of p16/Ki-67-positive CIN1 and CIN2 patients at baseline and after 3 months (3rd visit).

**Cytological findings–Baseline vs. 3rd Visit–Patients (*****n*****)**							**Difference in cervical smear findings–Patients (%)**				
**Cytology baseline**	**Patients (*n*)**	**NILM**	**ASC-US**	**LSIL**	**ASC-H**	**HSIL**	**Remission + Regression**	**Remission**	**Regression**	**Persistence**	**Progression**
**Active arm: vaginal gel**											
ASC-US	17	12	4		1		71%	71%		24%	6%
LSIL	40	25	5	7	3		75%	63%	13%	18%	8%
ASC-H	8	3	4		1		88%	38%	50%	13%	
HSIL	10	1	2	4	1	2	80%	10%	70%	20%	
Total	75	41	15	11	6	2	76%	55%	21%	19%	5%
**Control arm: watch and wait**											
ASC-US	20	4	5	5	6		20%	20%		25%	55%
LSIL	57	5	5	44	2	1	18%	9%	9%	77%	5%
ASC-H	15	3	2	3	6	1	53%	20%	33%	40%	7%
HSIL	5	1		1		3	40%	20%	20%	60%	
Total	97	13	12	53	14	5	25%	13%	11%	60%	15%

*Colors indicate different cytological changes: green: remission, light green: regression, yellow: persistence, red: progression. Remission: complete healing, Regression: change to a lower grade, Persistence: no change, Progression: change to a higher grade*.

*NILM, Negative for Intraepithelial Lesion or Malignancy; ASC-US, Atypical Squamous Cells of Undetermined Significance; LSIL, Low-grade Squamous Intraepithelial Lesion; ASC–H, Atypical Squamous Cells cannot exclude HSIL; HSIL, High-grade Squamous Intraepithelial Lesion*.

*Due to rounding, numbers presented may not add up precisely to the totals provided*.

A similar difference was still present at 6 months, which was mainly due to the disappearance of low-grade cytological findings (ASC-US and LSIL). 58/69 patients (84%) in the active arm and 37/96 patients (39%) in the control arm showed regression or remission (Table 3). The difference in regression rates between arms was statistically significant when analyzed as a dichotomous yes/no (Fisher's two-tailed exact test (*p* < 0.001) and after classification as remission, regression, persistence, and progression (Pearson Chi-squared test; *p* < 0.001).

**Table 3:** Cytological findings of the patients with p16/Ki-67-positive CIN1 and CIN2 at baseline and after 6 months (4th visit).

**Cytological Findings–Baseline vs. 4th visit–Patients (*****n*****)**							**Difference in cervical smear findings–Patients (%)**				
**Cytology baseline**	**Patients (*n*)**	**NILM**	**ASC-US**	**LSIL**	**ASC-H**	**HSIL**	**Remission + Regression**	**Remission**	**Regression**	**Persistence**	**Progression**
**Active arm: vaginal gel**											
ASC-US	16	11	4	1			69%	69%		25%	6%
LSIL	39	26	8	3	1	1	87%	67%	21%	8%	5%
ASC-H	6	4	2				100%	67%	33%		
HSIL	8	2	2		3	1	88%	25%	63%	13%	
Total	69	43	16	4	4	2	84%	62%	22%	12%	4%
**Control arm: watch and wait**											
ASC-US	19	5	6	5	3		26%	26%		32%	42%
LSIL	57	5	8	39	4	1	23%	9%	14%	68%	9%
ASC-H	15	4	4	6		1	93%	27%	67%		7%
HSIL	5	1	1	2	1		100%	20%	80%		
Total	96	15	19	52	8	2	39%	16%	23%	47%	15%

*Colors indicate different cytological changes: green: remission, light green: regression, yellow: persistence, red: progression. Remission: complete healing, Regression: change to a lower grade, Persistence: no change, Progression: change to a higher grade*.

*NILM, Negative for Intraepithelial Lesion or Malignancy; ASC-US, Atypical Squamous Cells of Undetermined Significance; LSIL, Low-grade Squamous Intraepithelial Lesion; ASC–H: Atypical Squamous Cells cannot exclude HSIL; HSIL, High-grade Squamous Intraepithelial Lesion*.

*Due to rounding, numbers presented may not add up precisely to the totals provided*.

*Fisher's two-tailed exact test and Pearson Chi-squared test/two-sample proportional test P <0.001 between the arms*.

The effect of the vaginal gel on cytological findings after 6 months flattened, but remained (**Figure 3**). Effects on regression and remission of low-risk cytological results after 6 months also persisted. The difference here from high-risk results was negligible (Table 3), while the positive effect on progression still remained after 6 months. The diminishing effect after 6 months in respect of high-risk results was probably due to cessation of the vaginal gel administration after 3 months.

**Figure 2:**
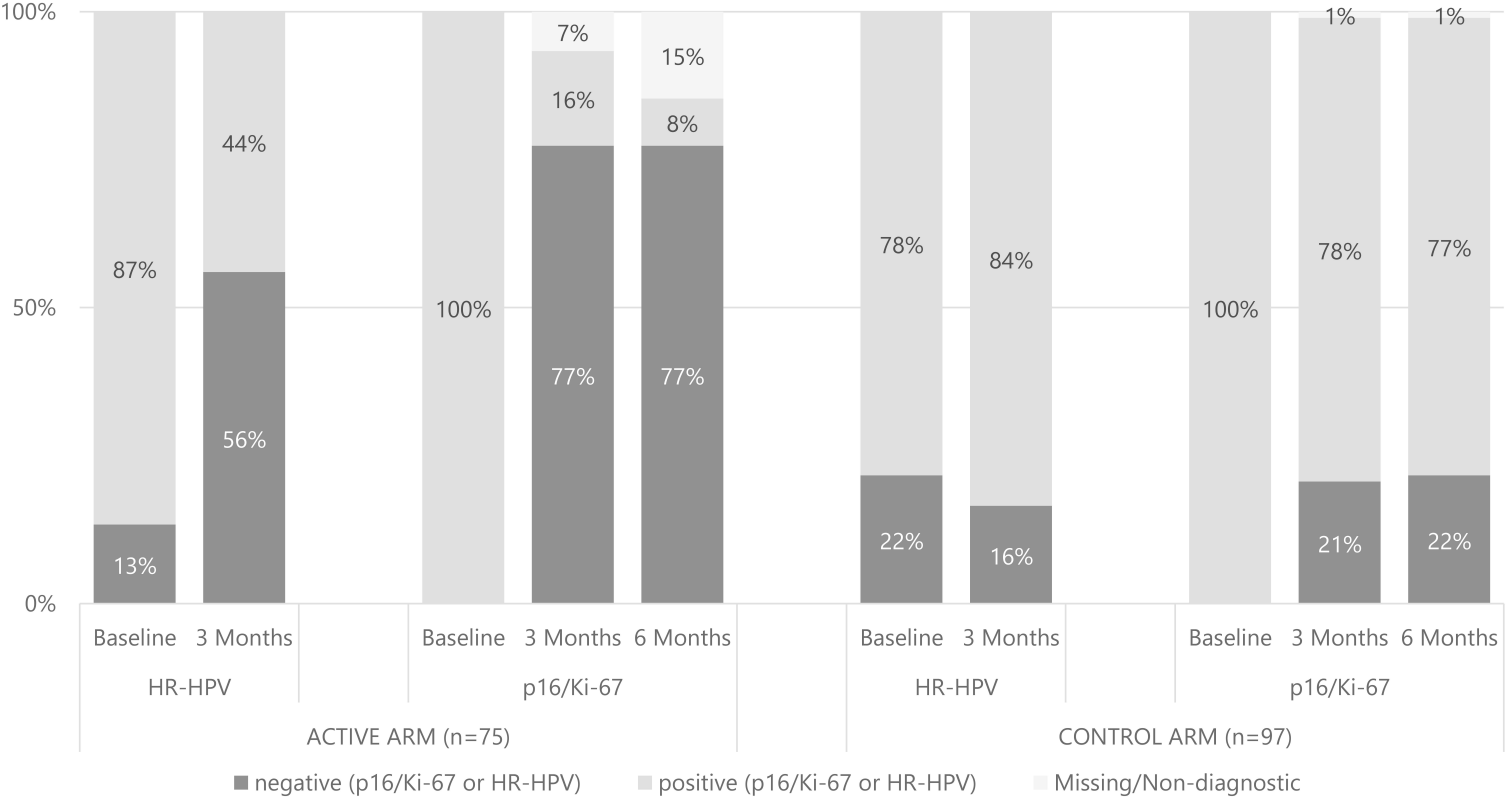
Correlation of high-risk HPV and p16/Ki-67 at baseline, 3 and 6 months in the active arm with vaginal gel vs. the control arm.

At the end of treatment, 77% of initially CINtec® Plus positive reverted to negative in the active arm, whereas only 21% in the control arm reverted negative after 3 months (Figure 2). The difference in the number of negative patients for the CINtec® Plus test was statistically significant in favor of the treatment group (Fisher's two-tailed exact test; *p* < 0.001).

At 6 months CINtec® Plus results were comparable to the 3-month data (Figure 2). In the active arm, 58 of 75 patients (77%) initially positive for CINtec® Plus became negative, whereas in the control arm 21 of 97 (22%) initially positive became negative. The difference in CINtec® Plus results between arms at 6 months was statistically significant (Fisher's two-tailed exact test; *p* < 0.001).

**Figure 3:**
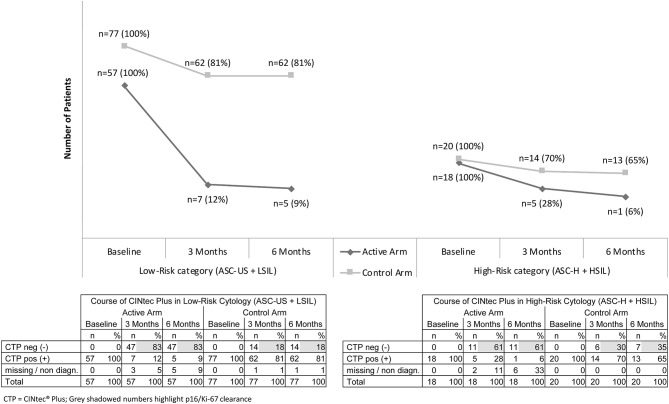
The evolution of Low-Risk (ASC-US & LSIL)* and High-Risk (ASC-H & HSIL)** patients at 3 and 6 months and the course of p16/Ki-67-positive and negative findings. *ASC-US: Atypical squamous cells of Undetermined significance; LSIL, Low grade squamous intraepithelial lesion. **ASC-H, Atypical Squamous Cells cannot exclude HSIL; HSIL, High-grade squamous intraepithelial lesion. *n* = number of patients (% of patients). Due to rounding, numbers presented may not add up precisely to the totals provided.

The effect of SAM gel on initially p16/Ki-67-positive patients was most prominent in the low-risk category (ASC-US and LSIL findings). After 3 months only 7 of 57 patients (12%) remained positive in the active arm, whereas 62 of 77 (81%) in the control arm were still positive for p16/Ki-67. This corresponds to a p16/Ki-67 clearance of 82% (47 of 57 patients) in the active arm compared to 18% (14 of 77 patients) in the control arm after 3 months in the low-risk category. Data from three patients (5%) were missing in the active arm and from one patient (1%) in the control arm (Figure 3).

A similar effect was also clearly visible in the high-risk category (ASC-H and HSIL findings). After 3 months only 5 of 18 patients (28%) remained positive for p16/Ki-67 in the active arm, vs. 14 of 20 (70%) in the control arm. This corresponds to a p16/Ki-67 clearance of 61% (11 of 18) in the active arm compared to 30% (6 of 20) in the control arm after 3 months in the high-risk category. In this category data from two patients (11%) were missing from the active arm (Figure 3).

At 6 months, 3 months after the end of treatment, the effect of SAM gel on p16/Ki-67 could still be observed in both low- and high-risk lesion groups (Figure 3).

Regarding hr-HPV (HPV 16 and 18, and 12 other hr-HPVs), 65 of 75 patients (87%) were tested positive for hr-HPV in the active arm at baseline (Figure 2). After 3 months of treatment, 33 patients (44%) were hr-HPV positive. In total, 42 of 75 (56%) of patients were negative for hr-HPV at 3 months, whereby clearance had occurred in 32 of 65 patients (49%); 10 of 75 patients (13%) were already negative for hr-HPV at baseline. No single patient was newly infected by hr-HPV in the active arm. In the control arm, 76 of 97 patients (78%) were positive for hr-HPV at baseline. At 3 months, 81 patients (84%) were hr-HPV positive (Figure 2). Eight of 76 patients (11%) were cleared from hr-HPV, whereas 13 were newly infected. The difference in hr-HPV prevalences between arms after treatment was statistically significant (Fisher's two-tailed exact test; *p* < 0.001).

As described in the randomized controlled trial, no major adverse events (AE) were observed and nobody in the minor AE required termination of device administration (14). Of the recorded 42 AEs, 12 reported by four patients were classified as possible/not known to be likely- or causally-related to the device (14). Most of the AEs were local (vaginal itching/burning, vaginal bloody discharge, increased vaginal bleeding, vaginal mycosis, or herpes) as well as slight abdominal pain or cramps. Nobody required termination of administration of the device. No serious possibly device-related adverse events occurred. Serum selenium measurement at the third visit (active arm only, 75 patients) confirmed that there was no systemic absorption of selenium (14).

## Discussion

The adsorptive and antioxidant vaginal gel significantly cleared hr-HPV and p16/Ki-67, a biomarker for oncogenic transformation. Treatment was associated with improved cytological outcomes in both low- and high-grade findings after 3 months of treatment. Cytological regression and p16/Ki-67 changes remained in the active arm (AA) at 6 months. To our knowledge this is the first demonstration of the efficacy of a non-destructive topical administration to the vagina and cervix of p16/Ki-67- and hr-HPV-positive women. It is the first study to utilize p16/Ki-67 as biomarkers and oncogenic hr-HPV. Prior to the parent trial (14), a retrospective data analysis conducted at the Sigmund Freud University, Vienna, Austria, established the safety and tolerability of this vaginal gel. That analysis too suggested a beneficial effect on abnormal cytological findings (23).

The present study demonstrates that the vaginal gel is both effective for the treatment of p16/Ki-67-positive abnormal cervical smears and for clearing hr-HPV. Gel administration led to significant treatment success in terms of increased cytological regression of precancerous disease in respect of 76% in the AA vs. 25% in the CA; and by significantly decreased progression by 5% in the AA vs. 15% in the CA. The significant decrease of hr-HPV (from 65 to 33 patients in the AA) and CINtec® Plus (from 75 to 12 positive tests) compared to the watch and wait CA were in line with cyto-pathological observations. The effect could still be observed in respect of regression (84% in the AA vs. 39% in CA) and progression (4% in the AA vs. 15% in CA) (Table 3) 3 months after the end of active treatment. Moreover, in women with low-risk cytology, gel treatment was associated with 82% p16/Ki-67-negative results after 3 months in the AA vs. 18% in the CA. High-risk lesions improved in 61% of patients in the AA compared with 30% in the CA (Figure 3).

CIN1 is the most frequent histopathology finding in cervical biopsies. It has a 12–16% progression rate to more advanced precancerous disease (15). Consequently, a clinical goal should be to treat only selected patients associated with oncogenic risk. Such a procedure should at the same time prevent progression and avoid future over-treatment. This should also decrease the rate of conisations and unnecessary destruction of lesions that may not have progressed. It is important to select cases of CIN1 with a specific biomarker, such as the tumor suppressor protein p16.

In this approach it is important to note that p16/Ki-67 has a high specificity for abnormal cytological findings and CIN. It is worth mentioning that ASC-US/LSIL had a specificity of 75.2% for CIN3 in p16/Ki-67-positive smears. The risk of CIN3 in hr-HPV- and p16/Ki-67-negative ASC-US/LSIL was 1.2%; in hr-HPV-positive smears it was 15.6%, and this increased to 27% in smears when the tests were also positive for p16/Ki-67 (24). In the follow-up of CIN2/3 patients treated by the LLETZ procedure (large loop excision of the transformation zone), additional testing for p16/Ki-67 resulted in improved specificity for recurrent CIN2+ than hr-HPV alone; 74.2 vs. 58.1% (17). In another study specificity for both p16/Ki-67 alone and for cytology was 95%, and for HPV alone it was 41.6% for detecting CIN2+/VAIN2+. Additionally, p16/Ki-67 was the best test for detecting cervical and vaginal lesions and to prevent under-diagnoses (18). In a study with ASC-US patients, the specificity of detecting high-grade dysplasia was highest for p16/Ki-67 immunocytochemistry, 74.2% in CIN3+ and 82.5% in CIN2+; significantly better than for HPV (19). p16/Ki-67 testing reduced referrals for colposcopy, and detected most CIN3 with high sensitivity and specificity compared with HPV in LSIL patients (20). Triage of women with p16/Ki-67 hr-HPV-positive cervical smears is superior than cytology and at the same time reduces unnecessary consultations, colposcopies and biopsies (21, 25, 26).

To our knowledge there are currently no published data concerning successful treatment of p16/Ki-67-positive CIN lesions. However, studies have been performed to investigate medical treatments for CIN1 and CIN2 lesions. Relatedly, Rahangdale et al. (27) applied 2 g of topical 5% 5-FU cream (Efudex; Valeant Pharmaceuticals International, Quebec, Canada) eight times over 4 months. Regression rates for CIN2 improved significantly; 93% in the 5-FU group compared to 56% in the observational group. However, 5-FU may induce vaginal adenosis after topical treatment in CIN1 (28). CIN1 and CIN2 patients, as in our study, were treated topically by Ashrafian et al. (29), in a randomized controlled trial with 3,3-di-indolylmethane (DIM), a stable metabolite of indole-3-carbinol (I3C). Two other randomized controlled trials are worth mentioning. One used three applications of cidofovir gel in a cervical cap before conisation and the other imiquimod with self-applied vaginal suppositories over 4 months (30, 31). Due to local and systemic adverse effects, imiquimod is inappropriate for treatment of CIN (32). A new trial with imiquimod for CIN2+ had to be stopped due to lagging inclusions (33).

Moreover, measurements of p16/Ki-67 suggested that SAM gel influences oncogenic progress and has therapeutic potential. Results indicate that alteration of the vaginal milieu by SAM gel over 3 months may reverse the oncogenic activity of hr-HPVs, at least temporarily. This may be mediated by alteration of the vaginal microbiome (34, 35).

The main advantage of this vaginal gel is its non-destructive effect and its suitability for administration during the watch and wait period when no other treatment options are available. The patient can administer the product herself and no clinic visit is necessary. Longer-term follow-up studies are necessary to evaluate the effect of SAM gel on the management of precancerous disease of the cervix.

## Limitations

One of the main limitations of the present study is that CIN2 and p16-positive CIN1 were not equally distributed between study arms. However, it is unlikely that this had an impact on the results and conclusions. A higher number of CIN2 and CIN1 IHC p16 findings, which correlate with lower spontaneous regression, were in the AA. As women in the AA showed higher regression compared to the CA, the therapeutic effect may even be higher than reported. However, before recommending a topical, non-destructive treatment to patients with CIN1/CIN2 in routine practice, positive study data should evaluate histological results as the final endpoint, as histology with biomarkers remains the gold standard. Since patients in the parent randomized study with abnormal colposcopy had a biopsy with histology and a biomarker analysis after treatment with the vaginal gel (14), the administration of an adsorptive and antioxidative vaginal gel could be recommended.

## Data Availability Statement

The original contributions presented in the study are included in the article/supplementary material, further inquiries can be directed to the corresponding author/s.

## Ethics Statement

The studies involving human participants were reviewed and approved by Multicenter Ethics Committee of the Faculty Hospital Olomouc and Medical Faculty of University Purkyně in Olomouc (Etická komise Fakultní nemocnice Olomouc a Lékarské fakulty UP v Olomouci, February 2017; Reference number: 9/17 MEK 1) and Local Ethics Committee of the University Hospital Motol, Praha, (Etická komise Fakultní nemocnice v Motole, October 2017; Reference number: EK-1278/17). The patients/participants provided their written informed consent to participate in this study.

## Author Contributions

Protocol development and manuscript writing were performed by ALM, EMG, and IM. Data collection and manuscript editing were performed by AS, VD, TM, and MP. All authors have read and agreed to the published version of the manuscript.

## Conflict of Interest

EMG was employed by company Phidalsa Pharma Consultants, Geneva, and by Camara & Partners Consultancy, Nyon, Switzerland. EMG and ALM have received fees for consulting and for redaction of the protocol draft from DEFLAMED International s.r.o. The remaining authors declare that the research was conducted in the absence of any commercial or financial relationships that could be construed as a potential conflict of interest.
